# COVID-19 Pandemic—Knowledge, Attitudes, Behaviours, and Actions among Faculty of Health Sciences Students

**DOI:** 10.3390/ijerph182212137

**Published:** 2021-11-19

**Authors:** Anna Stefanowicz-Bielska, Magdalena Słomion, Joanna Stefanowicz

**Affiliations:** 1 Department of Internal and Pediatric Nursing, Faculty of Health Sciences with Institute of Maritime and Tropical Medicine, Institute of Nursing and Midwifery, Medical University of Gdansk, 80-211 Gdansk, Poland; magdalena.slomion@gumed.edu.pl; 2 Department of Pediatrics, Hematology and Oncology, Faculty of Medicine, Medical University of Gdansk, 80-211 Gdansk, Poland; joanna.stefanowicz@gumed.edu.pl; 3 Faculty of Health Sciences with Institute of Maritime and Tropical Medicine, Medical University of Gdansk, 80-211 Gdansk, Poland

**Keywords:** COVID-19, knowledge, attitudes, behaviours, students

## Abstract

The aim of the study was to assess the level of knowledge about COVID-19 disease and preventive behaviour of undergraduate students of nursing, midwifery, and emergency medicine. Material and Methods: An electronic survey was conducted among students of nursing, midwifery, and emergency medicine during the COVID-19 pandemic, from 8 April 2021 to 6 June 2021 in the Pomeranian Voivodeship. Results: A total of 37 men and 238 women participated in the study. A moderate level of knowledge was found in 88% and high in 11% of students Midwifery students had a higher level than nursing students (*p* = 0.002) and students of emergency medicine (*p* = 0.003). The female gender is much more afraid of SARS-CoV-2 virus infection (*p* = 0.021). The most common preventive measure was to avoid people who coughed or had a cold (93%). Only 84% of students were vaccinated. Students who had a high level of knowledge more often avoided people who coughed or had a cold (*p* = 0.03) and gave up on meeting with friends (*p* = 0.02). Conclusions: Constant education of students on the principles of dealing with the risk of infection with SARS-CoV-2 is necessary. It is important to continually motivate students to adhere to the principles of prevention. In the face of the existing epidemiological threat, there is a need to change the curricula for the fields of health sciences by adding the subject infectious diseases—clinic and nursing, with special attention to practical aspects.

## 1. Introduction

At the end of December 2019, the first case of pneumonia attributed to Coronavirus Disease 2019 (COVID-19) was reported in the city of Wuhan in Hubei Province, China [1].

COVID-19 is caused by severe acute respiratory syndrome coronavirus 2 (SARS-CoV-2) [2]. The disease is highly contagious. Coronavirus infection affects all age groups, but elderly and chronically ill people are most at risk of severe disease [3]. Coronaviruses are most often spread by large droplets from the respiratory tract and by direct exposure; however, there are also other routes of transmission (i.e., aerosolsirborne and contact with infected surfaces) [4]. Symptoms of COVID-19 may appear 2–14 days after exposure to the virus [5]. Patients show a wide range of symptoms, such as fever, chills, cough, fatigue, headache, sore throat, diarrhoea, nausea and vomiting, loss of taste and smell, muscle or body aches, difficulty breathing and shortness of breath, and mucosal congestion or runny nose [5]. On 30 January 2020, the World Health Organization (WHO) declared COVID-19 a public health emergency of international concern [6].

According to the WHO situation report published on 23 July 2021, there were over 192,284,207 confirmed cases and 4,136,518 deaths worldwide. The same report found that the United States had the highest number of cases in the world, with an estimated 33,875,385 confirmed cases, including 604,546 deaths. In Poland, there were 2,881,948 confirmed cases of COVID-19 and 75,235 deaths [7]. By 25 July 2021, a total of 3,646,968,156 doses of vaccine were administered worldwide [7].

Health care professionals play an important role in the fight against COVID-19. Proper knowledge and appropriate behaviour regarding COVID-19 precautions among health care professionals are essential for their self-protection as well as effective disease control and prevention [8,9]. It is also very important for health sciences students. The education of students on the principles of dealing with SARS-CoV-2 infection is important independent of the country in which they study and live. The fight against COVID-19 is an international problem.

During the COVID-19 outbreak, health facilities needed help to deal with large numbers of COVID-19 patients. Many countries began to allow medical and health sciences students to perform certain health services. Moving forward, it is important to be sure the students are fully informed about the infection and how to protect themselves. The knowledge of Faculty of Health Sciences students about COVID-19 disease and prevention is very important because of their direct contact with patients during study. The main difficulty in the fight against COVID-19 is the fact that COVID-19 disease is a new disease and medical students must learn new rules of preventive behavior.

The aim of this study was to assess the level of knowledge about COVID-19 disease and preventive behaviour among undergraduate students in nursing, midwifery, and emergency medical services.

## 2. Materials and Methods

### 2.1. Design

This was a cross-sectional survey carried out during the COVID-19 pandemic from 8 April 2021 to 6 June 2021. Participation in the study was anonymous.

### 2.2. Participants

The participants of the study were full-time undergraduate students in nursing, midwifery, and emergency medical services. The study was conducted in all medical universities of the Pomeranian Voivodeship.

### 2.3. Questionnaire

An original questionnaire consisting of 31 questions was divided into five parts: Part 1—general characteristics of the participants (sex, field of study, and academic degree); Part 2—main source of information on SARS-CoV-2 virus and COVID-19 disease (Ministry of Health, Centers for Disease Control and Prevention, World Health Organization, European Centre for Disease Prevention and Control, social media, medical websites, scientific articles); Part 3—test-knowledge of the aetiology of SARS-CoV-2 (one question), routes of transmission of coronaviruses (one question), SARS-CoV-2 incubation time (one question), COVID-19 symptoms (one question), different ways of transmitting SARS-CoV-2 (one question), ways to prevent SARS-CoV-2 infection (five questions), and vaccinations (five questions); Part 4—students’ preventive behaviours (giving up meeting with friends, avoiding crowded places, avoiding visits to confined spaces, avoiding public transport, avoiding people who cough or have a cold, washing hands, disinfecting items that are touched frequently (e.g., door handles and surfaces), and talking to family and friends about ways to prevent COVID-19 (vaccination)); Part 5—students’ attitudes regarding fear of SARS-CoV-2 infection (“I’m afraid of contracting SARS-CoV-2”: strongly agree, agree, disagree, strongly disagree) and the degree of risk of SARS-CoV-2 infection (“I am at a higher risk of contracting COVID-19 than anyone else”: strongly agree, agree, disagree, strongly disagree) and self-assessment of the level of knowledge (“I believe that my knowledge of SARS-CoV-2 infection is”: low, medium, high). The knowledge test was developed by the authors of the study based on the available literature.

Knowledge was assessed with 15 statements about the aetiology and symptoms of COVID-19, SARS-CoV-2 transmission, and infection prevention. Each correct answer was worth one point. The maximum number of points was 15.

It was assumed that a level of ≤6 points from the test indicated a low level of knowledge, 7–11 points indicated average knowledge, and ≥12 points indicated a high level of knowledge.

### 2.4. Data Collection

The data were collected via an online questionnaire created in Google. A link to the survey was sent to all nursing, midwifery, and emergency medical services students via emails from the universities. Participation in the study was voluntary. The study was approved by the individual universities and approved by the Independent Bioethics Committee for Scientific Research at Medical University of Gdansk (NKBBN/364/2021).

### 2.5. Statistical Methods

For each parameter, the mean (X), median (M), standard deviation (SD, range (min, max), and lower and upper quartiles (25Q, 75Q) were calculated. Statistical significance between means for different groups was calculated by one-way analysis of variance (ANOVA), alternatively using the nonparametrical Mann–Whitney U test (for two groups) or Kruskal–Wallis test (for more than two groups), when the variances in groups were not homogeneous (the homogeneity of variance was determined by Levene’s test).

The statistical significance between frequencies was calculated by the chi-square test χ^2^_df_ with corresponding degrees of freedom df (df = (x^−1^) × (y^−1^), where x was the number of rows and y was the number of columns).

A *p*-value of less than 0.05 was required to reject the null hypothesis. Statistical analysis was performed using the EPIINFO Ver. 7.2.3.1 (Centers for Disease Control and Prevention in Atlanta, GA, USA) and Statistica Ver. 13.3. (TIBCO Software Inc. Palo Alto, CA, USA) software packages.

## 3. Results

### 3.1. Characteristics of the Participants

A total of 275 students took part in the study, including 37 men and 238 women. The mean age of all students was 21.5 (standard deviation ±3.83, with a range of 19–39). At the time of the survey, Pomeranian Voivodeship had 623 nursing students, 139 midwifery students, and 102 emergency medical services students. A total of 175 nursing students, 60 midwifery students, and 40 emergency medical services students responded to the survey, and 97 first-year, 88 s-year, and 90 third-year students participated.

### 3.2. Source of Information

The largest number of students (32%) reported that their main source of knowledge was information posted on the Ministry of Health website, followed by the World Health Organization website (19%), social media (19%), medical websites (15%), and scientific articles (15%). Only one person used the U.S. Centers for Disease Control and Prevention website.

### 3.3. The Knowledge Test

The results of the knowledge test for individual students were analysed. The median score was 10 points, the minimum score was 6 points, the maximum score was 13 points, the 25 Q was 9 points, and the 75 Q was 11 points.

None of the students scored the maximum number of points. Using the adopted scale, a low level of knowledge was found in two students, while the average score was 88% and the high score was 11%.

A total of 13/15 points were scored by 5 participants, 12/15 points by 26, and 11/15 points by 63.

The number of individuals with test scores accordingly is shown in Table 1. The most common result of the knowledge test was 9 correct answers (points).

Students gave the most correct answers to questions about the obligation to vaccinate against COVID-19 in Poland (98%), whether a history of SARS-CoV-2 infection is an absolute contraindication to COVID-19 vaccination (97%), aetiology of COVID-19 disease (97%), and the type of protective respiratory measures that should be used when caring for a patient with suspected or confirmed SARS-CoV-2 infection (96%).

The fewest correct answers were given in response to the questions about the incubation time of SARS-CoV-2 infection (17%), the type of hand disinfectants that are recommended in the prevention of coronavirus infection (25%), coronavirus transmission routes (31%), and the risk of severe SARS-CoV-2 infection in pregnant women (38%).

### 3.4. Preventive Behaviours

The survey addressed issues of prevention attitudes and behaviours in the era of the COVID-19 pandemic (Table 2).

A majority of respondents (93%) declared avoiding people who cough or have a cold. Less than half of students (41%) opted out of meeting with friends. Slightly more than half (53%) reported increasing the frequency of cleaning and disinfecting items that they touch often. A majority of students (84%) were vaccinated.

The statement of attitudes towards SARS-CoV-2 infection is shown in Table 3.

Most students estimated that they had an average level of knowledge (73%), while others reported a high level of knowledge (19%) and a low level of knowledge (7%).

### 3.5. Influence of Various Factors on the Level of Knowledge

Comparing the level of knowledge between females and males showed that sex made no difference (*p* = 0.682).

Analysing the results by field of study, it was shown that midwifery students have a higher level of knowledge than students of nursing (*p* = 0.002) and emergency medical services (*p* = 0.003) (Table 4).

The year of study had no influence on the results of the knowledge test (*p* = 0.945).

The level of knowledge did not depend on the declared source of knowledge (*p* = 0.285).

It was also shown that the level of knowledge does not depend on the level of anxiety declared by students (*p* = 0.210) or on their self-assessment (*p* = 0.891).

In contrast, students who believed that they were more likely to develop COVID-19 than anyone else had a higher level of knowledge (*p* = 0.049).

There was no evidence of an impact of the field of study on the prompt, “I am at a higher risk of contracting COVID-19 than anyone else” (*p* = 0.623) or the year of study (*p* = 0.304).

Members of the female sex were shown to be significantly more fearful of SARS-CoV-2 infection (*p* = 0.021). Self-rated anxiety related to SARS-CoV-2 infection did not depend on the field of study (*p* = 0.094) or the year of study (*p* = 0.203).

Knowledge level was shown to influence attitudes and behaviours towards SARS-CoV-2 infection (Table 5). Students who had a high level of knowledge significantly more often avoided people who coughed or had a cold and gave up on meeting with friends. Other attitudes, behaviours, and actions taken towards SARS-CoV-2 infection were independent of the knowledge level.

## 4. Discussion

COVID-19 is a serious epidemiological problem that causes many emotions. Polish students of nursing, midwifery, and emergency medical services presented many different attitudes towards the disease. Having direct contact with patients during practicum classes and apprenticeships, they were exposed to SARS-CoV-2 and COVID-19 more often than the general population.

The aim of this study was to assess the level of knowledge about COVID-19 and the preventive behaviours of undergraduate students in nursing, midwifery, and emergency medical services.

The research was conducted in the form of a questionnaire disseminated during a period of distance learning resulting from the epidemiological restrictions introduced by the Polish Ministry of Health.

A total of 175/623 (28%) nursing students, 60/139 (43%) midwifery students, and 40/102 (39%) emergency medical services students responded to the survey. There was a noticeably higher percentage of midwifery and emergency medical services students who participated in the survey.

Surveys on the knowledge of and attitudes towards SARS-CoV-2 infection and COVID-19 among students of nursing, midwifery, and emergency medical services have been conducted in several countries: Italy, Spain, India, Morocco, the Arab Emirates, Saudi Arabia, and Palestine.

### 4.1. Published Studies Varied in the Number of Respondents, Characteristics of the Participants and Structure of the Questionnaires Testing the Knowledge and Preventing Behaviours. This Was a Reason for the Difficulties in Comparing Studies

A total of 1226 nursing students studying at seven universities participated in the survey conducted in Saudi Arabia. The vast majority of students (99%) were aware of the pandemic, and their primary source of information was social media (71%). More than three-quarters of the students were confident that the government and the Ministry of Health had organizationally coped with the COVID-19 pandemic. The average knowledge test score was 9.85/12 (SD = 1.62, range = 0–12). The majority of students followed the prophylactic measures given in the survey, except for the procedure of washing hands with soap and water for at least 20 s after blowing the nose, sneezing, or coughing (39.2%) and the procedure of daily washing and disinfecting frequently touched surfaces (41.6%). Female gender, more years of study, and high academic achievement were associated with a high level of knowledge about COVID-19. Among the questions included in the survey, the most difficult question was the transmission route; eating and contacting wild animals may be a source of COVID-19 infection (47% gave the correct answer) [10].

It is noteworthy that, in our study, students also had difficulty answering correctly regarding the recommendations for hand disinfection to prevent SARS-CoV-2 infection (25% correct answers). Knowledge of the SARS-CoV-2 transmission routes was also low (31% correct answers).

A similar survey was conducted in Italy with 525 nursing students. The survey response rate was exceptionally high (99.3%). Most of the respondents were women (70%). A total of 37.5% of the participants were first-year students, 35.3% were second-year students, and 27.2% were third-year students. A total of 95.4% of female students showed a high level of knowledge, and 4.6% showed a very low level of knowledge. The Italian study compared knowledge, attitudes, and practical approaches between students of different years of study and showed a difference concerning only the practical approaches. A score of <9/13 correct responses indicated a low level of knowledge, whereas a score of ≥9/13 indicated a good level of knowledge [11].

The Spanish study involved 170 nursing students and 67 medical students. Most of the study participants were female (86.9%). The students came from 21 Spanish universities. A total of 76.8% of them had contact with patients infected with SARS-CoV-2. Their main sources of information were social media, the Ministry of Health website and other official organizations (88.2%), and radio and television (76%). The study compared nursing and medical students. Nursing students felt more prepared to treat patients with COVID-19 than medical students, and they were more likely to wear surgical masks when dealing with a patient infected with SARS-CoV-2 [12].

In our study, the main source of knowledge was information posted on the Ministry of Health website (32%), from the World Health Organization (19%), and on social media (19%).

The Palestinian survey was conducted with 218 nursing students from American Arab University and Al-Quds University. The mean age of the respondents was 22.10 ± 4.185 years. Of the 218 respondents, 65.6% were women. A total of 60.6% of the respondents were third-year students. A total of 46.8% of respondents showed a medium level of knowledge about COVID-19, and 38.5% of respondents had a high level of knowledge.

The easiest questions for students were those related to precautions (59.2% correct answers) and policies for dealing with suspected or infected persons (58.3% correct answers). The most difficult questions were those about the disease transmission routes (22.5% correct answers). Knowledge of transmission routes was low in our study (31%). Only 21.6% of students were convinced of the validity of compliance with the principles of COVID-19 prevention [13].

Polish students, on the other hand, presented mainly an average level of knowledge (88%), and a high level was found in only 11% of students. It is necessary to underline that the level of knowledge in Polish students is lower than in Palestinian students.

The survey from Oman involved 222 medical and nursing students, 55% of whom were medical students. The majority (59.9%) of the study participants were female. Total COVID-19 knowledge scores ranged from 5 to 24, with a mean score of 16.5 (SD = 4.2), which was 66% of the highest possible score. Medical students obtained a higher level of knowledge about COVID-19 (M = 17.9, SD = 3.6) than nursing students (M = 14.7, SD = 4.2, *p* < 0.01). Similarly, medical students presented greater practical skills (M = 45.1, SD = 4.1) than nursing students (M = 43.0, SD = 5.8, *p* < 0.01).

The majority (46.4%) of students stated that their first source of information about COVID-19 was social media and unofficial websites [14].

The Moroccan study involved 1216 nursing students. The majority of participants (95.6%) were 18 to 23 years old, and 77.4% of them were female. Their main sources of information about COVID-19 were social media (67.9%), television (12.3%), health professionals (7.9%), and family (5.1%). Approximately 82% of participants believed that SARS-CoV-2 spread through respiratory droplets from infected people. The most common clinical symptoms of COVID-19 according to study participants were fever (97.6%), dry cough (92.4%), dyspnoea (82%), and fatigue (74.9%). Most participants (94.8%) correctly identified the COVID-19 incubation period (up to 14 days). Almost all study participants (99.3%) knew that COVID-19 was caused by a virus. Over 56.6% were concerned about contracting COVID-19. Almost all participants reported that they often avoided crowded places. Approximately 93.4% of respondents declared frequent wearing of face masks when leaving the house, and 85.5% often maintained social distancing. However, only 47.4% reported that they washed their hands frequently [9].

In India, a survey was conducted to assess the level of knowledge and perception of COVID-19 among medical and health sciences students. A total of 97.95% (715/730) of students completed the survey. The mean age of the study participants was 21.81 ± 2.6 years. A high percentage of students came from pharmacy school (45.73%), followed by medicine (22.52%), physiotherapy (11.47%), nursing (9.37%), and dentistry (7.83%). Most of the participants had adequate knowledge, and approximately 18% had partial knowledge of the symptoms of severe COVID-19 cases. The students demonstrated positive perceptions of compliance with COVID-19 policies and control. Their main sources of information were social media (Facebook, WhatsApp, YouTube, Instagram) (n = 466, 65.17%), followed by news media (TV/video) (n = 149, 20.84%). The majority of students (91.61%) confirmed that WHO-recommended methods of preventing COVID-19, i.e., washing hands with an alcohol-based disinfectant, avoiding personal contact, and keeping a distance of at least 1 m (social distancing), are very important elements of disease prevention [15]. In the Indian survey, the high response rate to the survey is notable.

A similar study conducted in the Arab Emirates included 431 (56%) medical students, 117 (15%) nursing students, 49 (6%) pharmacy students, 45 (6%) physiotherapy students, 44 (5.5%) dentistry students, 21 (3%) radiology students, and 66 (8.5%) students in other medical specialties. A total of 712 surveys were collected. Most respondents (87%, n = 647) obtained COVID-19 information from multiple sources; 7% (n = 52) from social media; and 6% (n = 48) from medical platforms, health care professionals, government media, or university newsletters. Seventy-six percent (n = 539) of participants did not recognize the correct routes of COVID-19 transmission, although most respondents correctly recognized its symptoms, mean incubation period, best diagnostic test, and its progression (95%, 85%, 89%, 89%, and 70%, respectively). The majority of respondents were aware of COVID-19 preventive measures, including methods to reduce the spread of the virus, isolation of positive cases, use of the N95 mask limited to health care workers, and the need for preventive measures among young adults and children (83%, 92%, 84%, and 87%, respectively). There was a highly significant difference in the overall scores representing COVID-19 knowledge between medical students and students of related subjects (median 17.5, 8.5–23, versus 16.5, 5–22.5, *p* < 0.0001, Mann–Whitney U test). Sixty-three percent (n = 431/686) of study participants were concerned about COVID-19 infection, while the vast majority (92%, n = 633/686) were concerned that a family member might contract the virus.

Sixty percent (n = 407/677) of students did not attend family gatherings and did not visit shopping malls, cafes, industrial areas, hospitals, or COVID-19 facilities as part of their volunteer work. Ninety-seven percent of subjects (n = 655) took precautions when taking home deliveries, 94% (n = 637) washed their hands more frequently, and 95% (n = 643) wore face masks [16].

The results of our study are consistent with those of other authors. Students present a comparable level of knowledge. The knowledge of health sciences students should be improved to have better prevention of the SARS-CoV-2 infection.

An interesting aspect of the research is comparing the knowledge of students of particular faculties. In studies from Omam, India, and the Arab Emirates, the level of knowledge of medical students and students of health sciences was compared, showing a higher level of knowledge among medical students [14,15,16]. An interesting aspect of our study is comparing the knowledge test scores of the different health sciences faculties and finding that midwifery students possessed a higher level of knowledge than nursing and emergency medical services students.

Perhaps the knowledge test scores would have been higher if the teaching had been done in a classroom setting rather than remotely.

### 4.2. Study Limitations

The main limitations of this study were as follows: conducting the study during the distance learning period, performing it only in one region of Poland, and the lack of statements in the questionnaire about methods of prevention used during meetings of friends.

## 5. Conclusions

Constant education of students on the principles of dealing with the risk of SARS-CoV-2 infection is necessary. It is important to continually motivate students to adhere to the principles of prevention. In the face of the existing epidemiological threat, there is a need to change the curricula for the fields of health sciences by adding the subject infectious diseases—clinic and nursing, with special attention to practical aspects.

## Figures and Tables

**Table 1:** Knowledge test results for students of nursing, midwifery and emergency medical services.

Number of Correct Answers/Points	Number of Students
6	2 (0.73%)
7	18 (6.55%)
8	29 (10.55%)
9	76 (27.64%)
10	56 (20.36%)
11	63 (22.91%)
12	26 (9.45%)
13	5 (1.82%)

**Table 2:** Preventive behaviour among students of nursing, midwifery, and emergency medical services.

Preventive Behaviours	Number of Affirmative Answers
1. I gave up meeting with friends.	114 (41.45%)
2. I limited my use of public transportation.	163 (59.27%)
3. I limited my visits to confined spaces (e.g., I rarely go shopping, to the library, or to church).	213 (77.45%)
4. Whenever possible, I avoid people who cough or have a cold.	257 (93.45%)
5. I avoid places where large numbers of people gather.	235 (85.45%)
6. I increased the frequency of cleaning and introduced disinfection of items that are frequently touched (e.g., door handles and surfaces).	147 (53.45%)
7. I wash my hands more often than usual.	235 (85.45%)
8. I have explained to family and friends the ways to prevent COVID-19.	236 (85.82%)
9. I have been vaccinated.	232 (84.36%)

**Table 3:** Attitudes of nursing, midwifery, and emergency medical services students towards SARS-CoV-2 infection.

Attitudes towards SARS-CoV-2 Infection	Strongly Agree	Agree	Disagree	Strongly Disagree
I’m afraid of contracting SARS-CoV-2 virus.	27 (9.82%)	131 (47.64%)	96 (34.91%)	21 (7.64%)
I am at a higher risk of contracting COVID-19 than anyone else.	14 (5.09%)	59 (21.45%)	126 (45.82%)	76 (27.64%)

**Table 4:** Level of knowledge of nursing, midwifery, and emergency medical services students by field of study.

Field of Study	Number of Correct Answers in the Knowledge Test				
	*N*	Average	SD	Min	Max
Nursing	175	9.63	1.47	6.00	13.00
Midwifery	60	10.33	1.27	7.00	13.00
Emergency medical services	40	9.48	1.47	7.00	12.00

ANOVA *p* = 0.002. Nursing vs. midwifery *p* = 0.002. Nursing vs. emergency medical services *p* = 0.552. Midwifery vs. emergency medical services *p* = 0.003.

**Table 5:** Attitudes, behaviours, and actions taken towards SARS-CoV-2 infection by nursing, midwifery, and emergency medical services students and their level of knowledge.

Attitudes, Behaviours, and Actions Taken towards SARS-CoV-2 Virus Infection		Low Level of Knowledge	Average Level of Knowledge	High Level of Knowledge	*p*
		% of Affirmative Responses			
		*N* (%)	*N* (%)	*N* (%)	
I’m afraid of contracting SARS-CoV-2.	strongly disagree	1 (4.76%)	19 (90.48%)	1 (4.76%)	0.210
	disagree	0	88 (91.67%)	8 (8.33%)	
	agree	1 (0.76%)	112 (85.50%)	18 (13.74%)	
	strongly agree	0	23 (85.19%)	4 (14.81%)	
I am at a higher risk of contracting COVID-19 than anyone else.	strongly disagree	0	71 (93.42%)	5 (6.58%)	0.049
	disagree	1 (0.79%)	109 (86.51%)	16 (12.70%)	
	agree	0	52 (88.14%)	7 (11.86%)	
	strongly agree	1 (7.14%)	10 (71.43%)	3 (21.43%)	
I gave up meeting with friends.		1 (50%)	93 (38.43%)	20 (64.52%)	0.020
I limited my use of public transportation.		2 (100%)	141 (58.26%)	20 (64.52%)	0.401
I limited my visits to confined spaces (e.g., I rarely go shopping, to the library, or to church).		2 (100%)	186 (76.86%)	25 (80.65%)	0.666
Whenever possible, I avoid people who cough or have a cold.		1 (50%)	226 (93.39%)	30 (96.77%)	0.030
I avoid places where large numbers of people gather.		1 (50%)	205 (84.71%)	29 (93.55%)	0.152
I increased the frequency of cleaning and introduced disinfection of items that are frequently touched (e.g., door handles and surfaces).		1 (50%)	128 (52.89%)	18 (58.06%)	0.859
I wash my hands more often than usual.		1 (50%)	205 (84.71%)	29 (93.55%)	0.152
I have explained to family and friends the ways to prevent COVID-19.		1 (50%)	208 (85.95%)	27 (87.10%)	0.341
I’ve been vaccinated.		1 (50%)	205 (84.71%)	26 (83.87%)	0.403

## Data Availability

Data available on request due to privacy restrictions.
